# Umbilical-spinous line: a morphological term that should be included in the anatomical terminology

**Published:** 2013-09-30

**Authors:** Jorge Eduardo Duque, John Ríos

**Affiliations:** 1 Department of Basic Sciences, School of Medicine. Universidad de Caldas. Manizales, Colombia Email: jduqueparra@yahoo.com.mx; 2 Department of Basic Biological Sciences, Universidad Autónoma de Manizales. Manizales, Colombia. Email: jhon.barco@ucaldas.edu.co

**Keywords:** Anatomy, vermiform appendix, appendicitis, umbilical-spinal line, McBurney point, anatomical terminology.

## Abstract

We argue the need to include in the International Anatomical Terminology the term *"Umbilical-spinous line"* for its importance as a morphological referent in bioscopic and surface anatomy. Also, in order to avoid using eponyms, it is suggested that the traditional term "McBurney point" be replaced by *"supra spinous point"* as being more descriptive of location.

## Abstract (es)

Se argumenta la necesidad de incluir en la Terminología Anatómica Internacional el término "*línea umbilico-espinosa*" por su importancia como referente morfológico en la anatomía bioscópica y de superficie. Además, con el propósito de evitar la utilización de epónimos, se sugiere que el tradicional término "*punto de McBurney*" sea remplazado por el de "*punto supraespinoso"*, por ser más descriptiva su ubicación.

In the current Anatomical Terminology, the official book of the Federated International Committee on Anatomical Terminology (FICAT), "*umbilical-spinous line*¨ does not appear to be named as an anatomical detail of the human body. The only structures that appear in the text related to the navel are: artery, fascia, and fissure, left side branch of the portal vein, umbilical region, umbilical ring and umbilical vein^1^.

The "*umbilical spinous line*" is an imaginary line traveling from the umbilicus to the right anterior superior iliac spine and concerning it is included the traditional term, "McBurney point", which corresponds to the point that defines the lateral and middle third of that line. At that point and depth, it lies at the base of the vermiform appendix^2^ (see Fig.1).

**Figure 1:**
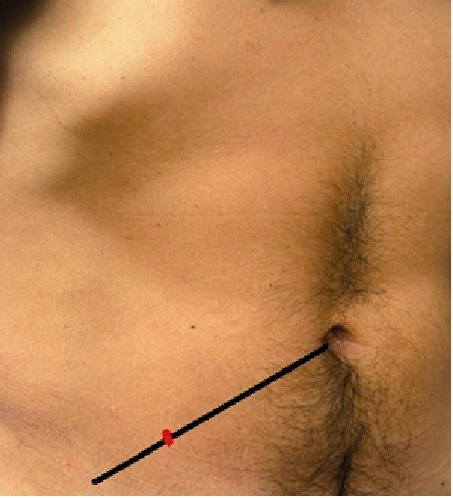
The black line demarcates the umbilical spinous line. The red mark indicates McBurney´s point.

McBurney's point was described in 1889 by Professor Charles Heber McBurney (1845-1913), who was a teaching assistant in Anatomy at the College of Physicians and Surgeons of Columbia University, New York, and continued in this position until his appointment as Professor of Surgery in 1889^3^. McBurney's notoriety is associated with the diagnostic sign of inflammation of the vermiform appendix and the surgical technique for the management of appendicitis. He reported that the palpation point of maximum sensitivity is determined by pressure being applied with a finger (McBurney's sign), and that this point is located between one and one-half to two inches from the right anterior superior iliac spine (McBurney point) on a straight line drawn from the spinous process to the navel^3^ ^,^ ^4^. This point corresponds to the union of the lateral third with the middle third of the umbilical spinous line^5^.

The location of the vermiform appendix usually has some anatomical variations, which explains why this structure cannot always be located at the McBurney´s point^3^. For over 100 years this surface mark^5^ has been used to locate the cecal appendix^5^ ^,^ ^6^; however, in a study carried out on 291 women of reproductive age, including pregnant and non-pregnant women, the location of the vermiform appendix in relation to that point was evaluated and it was determined that the location of the appendix is normal when it is within a range of 2 cm from McBurney´s point; outside of that range, it is considered that there is anatomical variation from change of position; additionally, no changes in the location of the appendix were observed in pregnant women ^6^.

For health professionals it is important to know this morphological detail of the abdominal-pelvic region for its association with acute appendicitis, a frequent surgical emergency in the world and for which it is estimated that 7% of the population will suffer from it at some point in their life^7^. Although there are cases where the presentation of this disease is atypical, many patients present with semiological characteristics of pain that finally is located at the level of the right iliac fossa, which is determined by palpation of McBurney´s point ^8^.

In other studies carried out to prove its validity and a study based on 275 double contrast radiographies with barium enemas found that only 35% of the bases of the appendices were found in the 5 cm range of McBurney´s point, while 15% were at more than 10 cm away in distance. These findings are consistent with global studies conducted by the World Gastroenterology Organization, which showed that least than half of all patients with appendicitis have maximum sensitivity on McBurney´s point ^5^.

Nevertheless, we must remember that many years ago -1895 - Germany established the first committee charged with pointing out over 5,000 anatomical terms with only one name, which constituted the Basilean Anatomical Catalog^9^, after which others followed. In 1933 it was decided to formally remove eponyms from terminology^10^. The XIII International Congress held in Rio de Janeiro in 1989 established the Federated International Committee on Anatomical Terminology^9^ (FICAT) which is the body responsible for ensuring that the majority of anatomical structures are named with a single word, that each anatomical term is as accurate and descriptive as possible and that eponyms are not used^9^. All this is intended to facilitate the teaching-learning process and also allow clear and accurate communication between all professionals and researchers in the area of ​​health^11^ ^,^ ^12^.

Based on foregoing considerations, and assuming that all professionals in the field of health accept the current Anatomical Terminology for describing not only physical structures but also conditions that affect the patient for medical or surgical evaluation, understanding would be easier because they would be communicating in terms of structure and function which does not happen when eponyms are used^13^ since applying a researcher's name to a given structure tells us nothing about its nature. In scientific language precision and clarity are important for the terms used since precision requires sharply defined scientific terms for meaning, while clarity is achieved when in a given context each term can be exclusively applied only to one object or phenomenon^14^.

It is a fact that every change initially generates a certain amount of resistance, especially with doctors and surgeons rooted in a culture in the management of a particular language loaded with eponyms with the risk of intoxicating themselves with this inappropriate symbolism that rather than approaching truth takes us further away from it ^15^. The only major obstacle standing in the way of human beings is language because it is easier to corrupt a written text than the memory of a surgeon, especially if he has learned a million words and does not dare to modify a single one for fear of losing the rest ^16^.

However, despite this reluctance, in recent years there has been increasing consciousness of the need to modify medical language, replacing eponyms for more descriptive terms to make communication more clear and precise. Thus, a large group of professionals in the medical field throughout Latin-America meet regularly at the Ibero/Latin American Symposium on Terminology - SILAT - to review, discuss and suggest changes in terminology, which are subsequently sent to FICAT for final study^17^. But more importantly, all members of SILAT come from years of teaching and transmitting disciplinary knowledge to new generations of physicians based on current terminology, so that language change, although slow, is still occurring.

Returning to the topic at hand and consistent with anatomical terminology, in the study of elements associated with the navel it was found that the umbilical region is highly significant and is taken into account by all texts for teaching macroscopic anatomy and in articles dealing with the clinical implications of appendicitis. Therefore, there is a need to include a new term in anatomical terminology, "*umbilical spinous line*", which would serve as a morphological referent for location on the given line, a specific point of auscultation of the vermiform appendix known from years ago by the eponym of McBurney´s point. Furthermore, in order to avoid using unacceptable eponyms in anatomical terminology, we suggest that the traditional McBurney's point be replaced by the "supraspinatus point."
